# Different facets of addiction from a systemic perspective: a case report

**DOI:** 10.1192/j.eurpsy.2024.821

**Published:** 2024-08-27

**Authors:** A. Osiak, U. Altunoez, M. Anthoff, G. Rietig, M. Bald, S. Akbas, I. T. Graef-Calliess

**Affiliations:** KRH Wunstorf Psychiatric Hospital, Wunstorf, Germany

## Abstract

**Introduction:**

Many individuals who suffer from one or multiple substance use disorders often also struggle with another co-morbid psychiatric condition, primarily anxiety disorders. As substance dependency develops, the loss of self-control becomes a pivotal issue, leading to challenges in self-esteem and self-respect. These challenges subsequently give rise to problems in interpersonal communication and close relationships. In addition to the biological model of substance use problems and highly manualized approaches, it is crucial to have a systemic understanding of the patient’s situation and appropriate treatment settings. This understanding allows for the proper consideration of individual differences and requirements, ultimately enabling better treatment and prevention strategies.

**Objectives:**

Our aim is to present a comprehensive case that shows various facets of addiction and introduce a systemic therapy concept of an integrative/systemic day clinic from Germany.

**Methods:**

Through a detailed case presentation, we will introduce a systemic-psychotherapeutic day-clinic concept from a psychiatric training hospital in Wunstorf, Germany.

**Results:**

Case: In the case of a 48-year-old female patient initially diagnosed with recurrent depressive disorder, it was later revealed that she also deals with alcohol addiction and its interconnectedness with sex addiction. Shame and a lack of self-esteem in relationships played a central role in her journey. She does not perceive herself to be loved but to be harmed, which led to many violent sexual acts that increased her feelings of shame. Systemic therapeutic approaches like family constellations were applied, helping her to experience her child-ego states. This profound insight propelled her willingness to change. She began prioritizing self-care, learning to like, accept, and eventually love herself. Aromatherapy aided in calming and maintaining focus. The patient learned to redefine her emotions, aligning them with reality, thereby enabling the adaptive fulfillment of her needs.

**Conclusions:**

Our interdisciplinary team at the day clinic employs therapeutic approaches like dynamic, cognitive-behavioral, and systemic therapy to thoroughly understand the patients and their conditions. This case underscores the significance of an individually tailored treatment drawing from diverse therapeutic concepts, especially in patients with addiction. The combination of different therapeutic approaches facilitates a profound engagement with the patient, potentially resulting in more intensive therapeutic work and a higher rate of success which should be evaluated in future studies.

**Disclosure of Interest:**

None Declared

